# Transthyretin Amyloidosis: From Mechanisms to Treatment

**DOI:** 10.3390/biom16101401

**Published:** 2026-09-25

**Authors:** Filippos Triposkiadis, Andrew Xanthopoulos, Georghios P. Georghiou, Anastasis Stephanou, Ioannis Patrikios

**Affiliations:** 1 Department of Cardiology, University of Thessaly, Biopolis, 411 10 Larissa, Greece; 2 School of Medicine, European University Cyprus, Nicosia 2404, Cyprusi.patrikios@euc.ac.cy (I.P.); 3 Department of Cardiothoracic Surgery, Aretaeio Hospital, Nicosia 2414, Cyprus; 4 Gray Faculty of Medical & Health Sciences, Tel Aviv University, Tel Aviv 6997801, Israel

**Keywords:** transthyretin amyloidosis, amyloid fibrils, kinetic stabilization, gene silencing, gene editing

## Abstract

Transthyretin amyloidosis (ATTR) is a progressive systemic disorder driven by the misfolding of transthyretin (TTR) proteins and their deposition as amyloid fibrils. It manifests as hereditary ATTR (ATTRv), caused by pathogenic variants, and wild-type ATTR (ATTRwt), linked primarily to aging. These deposits preferentially affect the myocardium and peripheral nerves, producing severe cardiomyopathy and polyneuropathy. The amyloidogenic cascade is initiated by the dissociation of TTR tetramers into misfolded monomers that aggregate into insoluble fibrils. Historically, treatment was limited to supportive care or organ transplantation; however, it has been transformed by TTR stabilizers, RNA interference (RNAi) agents, and antisense oligonucleotides (ASOs). More recently, CRISPR-Cas9 genome editing has emerged as a potential single-dose strategy for durable TTR suppression. In parallel, anti-fibril therapies, including amyloid-depleting monoclonal antibodies designed to clear existing tissue deposits, are being developed as a complementary disease-modifying strategy. This review delineates the molecular mechanisms of amyloidogenesis and evaluates current clinical management strategies. Recent advances in noninvasive diagnostics and targeted molecular therapies, enabled by precision medicine, have markedly improved disease management and clinical outcomes.

## 1. Introduction

Amyloidoses are diseases characterized by extracellular deposits of amyloid, a fibrillar material formed from precursor proteins [1]. Transthyretin (TTR) amyloidosis (ATTR) encompasses two discrete clinical entities: hereditary ATTR amyloidosis (ATTRv) [2] and wild-type ATTR amyloidosis (ATTRwt), that has been historically designated as senile systemic amyloidosis [3]. ATTRv is an autosomal dominant, progressive disorder originating from pathogenic mutations within the TTR gene [4]. To date, over 200 TTR sequence variants have been identified, encompassing pathogenic, likely pathogenic, benign, and variants of uncertain clinical significance. A significant proportion of these variants is associated with hereditary ATTR amyloidosis, exhibiting substantial heterogeneity in terms of penetrance, organ involvement, age of onset, and geographic distribution [5]. The phenotypic spectrum extends from ATTR variants predominantly associated with cardiomyopathy (CM) to those primarily characterized by polyneuropathy (PN) [6]. Conversely, ATTRwt is characterized as an age-dependent condition in which amyloid fibrils are formed from non-mutated TTR proteins [7,8]. Patients diagnosed with ATTRwt are typically elderly males (>90%), generally over 70 years of age, who frequently present with CM as well as orthopedic and musculoskeletal manifestations [9]. ATTR, regardless of its cardiac or neurological manifestations, is recognized as a progressive, severely debilitating, and ultimately fatal condition [10]. Amyloid CM is common in heart failure (HF). A meta-analysis of 11 studies involving 3303 patients reported a 13.7% prevalence, primarily driven by ATTR [11]. Furthermore, amyloid CM and aortic stenosis (AS) coexist in 6% to 16% of cases, with higher rates observed in elderly men undergoing transcatheter aortic valve replacement (TAVR) for severe AS [12]. Although systemic ATTR predominantly spares the central nervous system (CNS), certain pathogenic variants (e.g., Ala45Thr, Tyr69His, Gly53Arg) can produce a distinct leptomeningeal phenotype in which amyloid deposits accumulate in the leptomeninges and cerebral vasculature, causing recurrent intracranial hemorrhage, seizures, ataxia, and progressive cognitive decline [13,14]. Because CNS-penetrant therapies remain limited and hepatic-only interventions (e.g., liver transplantation, hepatic TTR silencing) do not address leptomeningeal deposition, this phenotype requires distinct diagnostic vigilance and dedicated therapeutic strategies [15].

Therapeutic advancements in ATTR have improved clinical management and patient prognosis through targeted disease-slowing mechanisms [16]. This manuscript aims to synthesize the current understanding of the molecular pathogenesis of ATTR, with particular emphasis on contemporary medical therapeutic modalities.

## 2. TTR Protein

TTR, or prealbumin, is a homotetrameric carrier protein for thyroxine and retinol-binding protein (RBP) [17]. Primarily synthesized by the liver, choroid plexus, and retinal epithelium, TTR circulates in the serum, cerebrospinal fluid (CSF), and ocular tissues [18].

### 2.1. Structure

**Figure 1:**
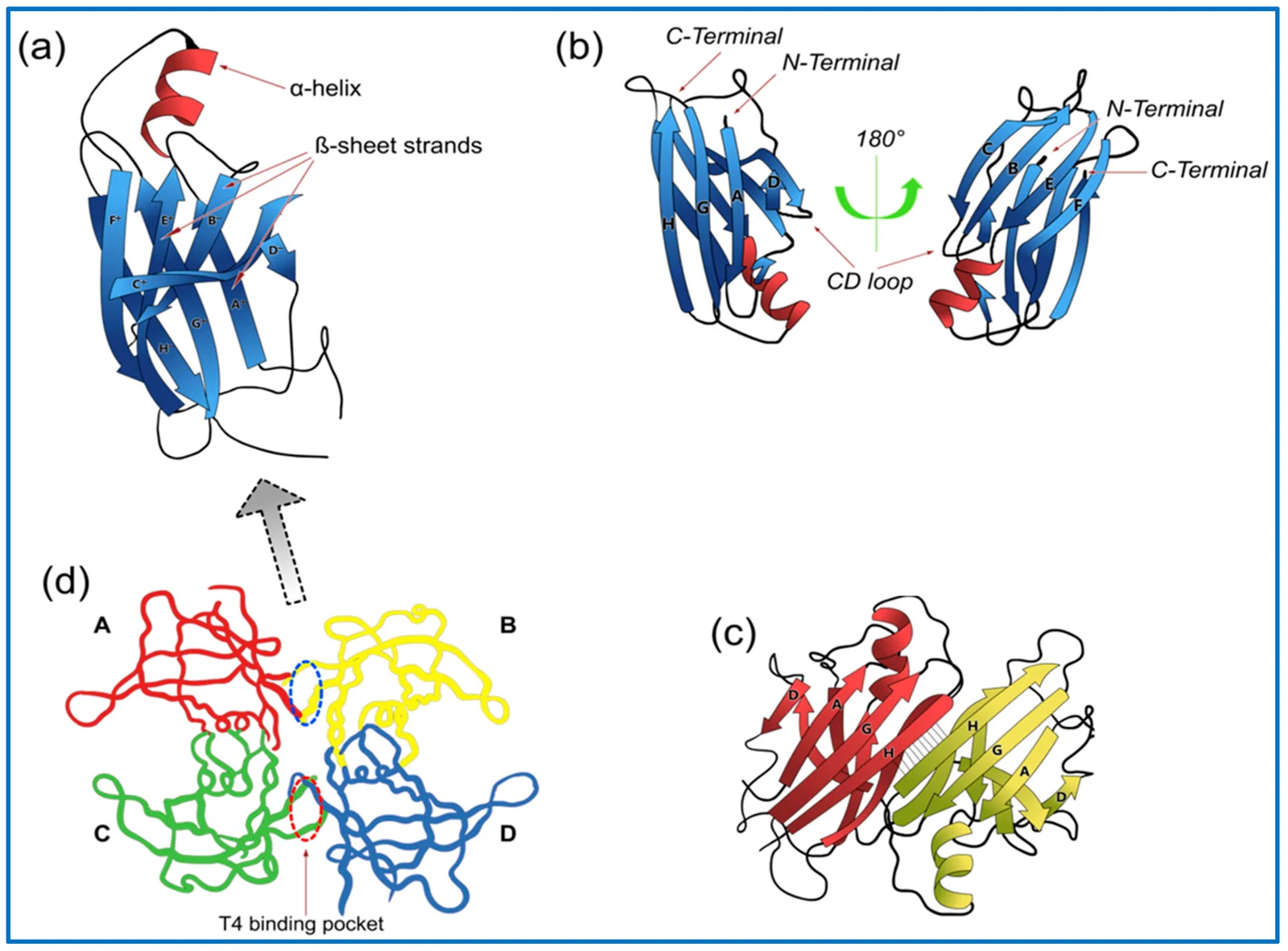
Transthyretin (TTR) structures. TTR is a homotetramer of four 127-amino-acid monomers. Each monomer features eight β-strands and one α-helix (**a**,**b**). Monomers bond via β-strands H–H’ and F–F’ to form dimers (**c**), which assemble into a tetramer through hydrophobic loop contacts. This tetramer contains a central channel with two hormone-binding sites (**d**). Adapted from Ref. [16].

Mature TTR is composed of four identical subunits, termed monomers (Figure 1) [19]. The TTR monomer is a globular protein composed of 127 amino acids (AAs) folded into eight β-strands [20]. These strands form two antiparallel β-sheets (DAGH and CBEF) that create a characteristic β-sandwich configuration. While the strands are connected by short loops, a prominent EF helix-loop motif exists between strands E and F. In the quaternary structure, this region forms a solvent-exposed interface that binds RBP for vitamin A transport [21]. Additionally, the DAGH sheet edge enables antiparallel hydrogen bonding between monomers, facilitating stable homodimer formation.

**Figure 2:**
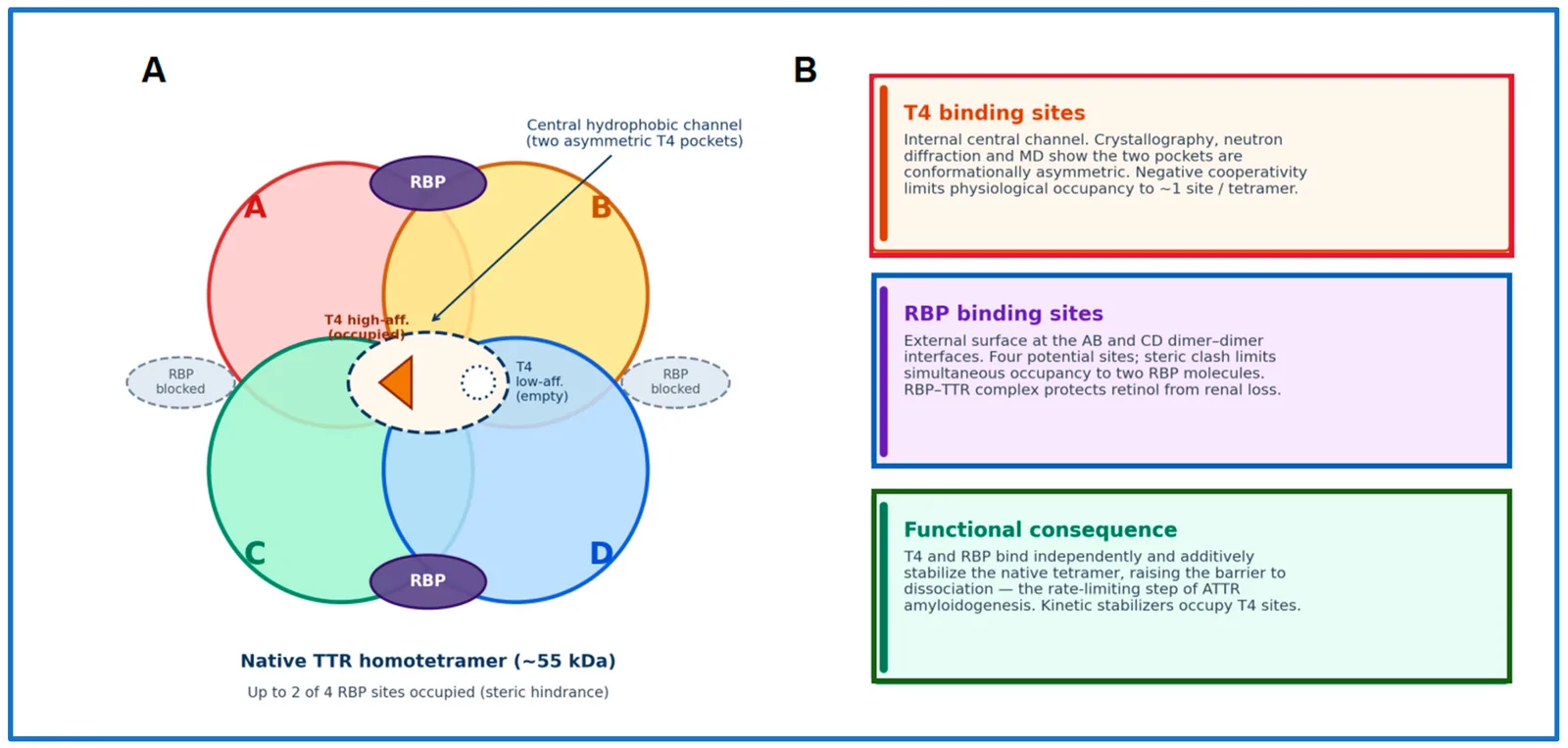
Transthyretin (TTR) tetramer: thyroxine (T4) and retinol-binding protein (RBP) binding sites. (**A**) Schematic of the native homotetramer (~55 kDa) assembled from subunits A–D. The two T4 binding sites lie in the central hydrophobic channel. Although long presumed to be structurally equivalent by symmetry, crystallographic and neutron-diffraction studies, together with molecular-dynamics simulations, show that extensive molecular motions render the pockets conformationally asymmetric, with one site typically binding ligand more tightly than the other. Negative cooperativity further limits physiological occupancy to approximately one T4 molecule per tetramer; the higher-affinity pocket is shown occupied and the lower-affinity pocket vacant. RBP (retinol-binding protein) binds the external surface at the AB and CD dimer–dimer interfaces. Four potential RBP sites exist, but steric hindrance restricts simultaneous occupancy to two molecules (occupied sites in purple; blocked sites dashed). (**B**) Functional consequences of occupancy. T4 and RBP bind independently and additively to stabilize the native tetramer, raising the kinetic barrier to subunit dissociation—the rate-limiting step of TTR amyloidogenesis. Kinetic stabilizers (tafamidis, acoramidis, diflunisal, tolcapone) act by occupying the T4 channel. ATTR, TTR amyloidosis. Created in BioRender. Triposkiadis, F. (2026) https://BioRender.com/jpp84e0.

The TTR quaternary structure forms a cylindrical shape with a central channel [22]. Assembly typically proceeds through a stable dimeric intermediate. The wild-type tetramer, weighing approximately 55 kDa, maintains structural stability under physiological conditions, preventing dissociation. The TTR tetramer contains two T4 binding sites within the central channel that exhibit negative cooperativity (Figure 2); although long presumed to be structurally equivalent by symmetry, crystallographic and neutron diffraction studies, together with molecular dynamics simulations, have shown that extensive molecular motions render the two sites conformationally asymmetric, with one pocket typically binding ligands more tightly than the other [23,24,25]. Initial binding reduces affinity for subsequent ligands; specifically, the first T4 molecule binds to the higher-affinity pocket. This occupancy induces negative cooperativity, significantly decreasing the binding affinity of the second, weaker site [26]. Consequently, under physiological conditions, typically only one site per TTR tetramer is occupied. This negative cooperativity enables TTR to function as a physiological buffer. Since fewer than 1% of sites are normally occupied, the vacant sites can sequester excess T4 during spikes, preventing toxicity while maintaining steady hormone release.

Unlike T4, which binds internally to TTR tetramers, RBP attaches to their external surface (Figure 2). Each TTR tetramer has four potential RBP binding sites (two per dimer). A maximum of two RBP molecules can bind, as steric hindrance prevents interaction with the remaining sites [27]. The binding of T4 and RBP stabilizes TTR tetramers and inhibits amyloidogenesis. In vitro studies confirm that RBP and T4 independently and additively prevent TTR tetramer disassembly [28].

### 2.2. Function

TTR protects and supports diverse organ systems. TTR plays a pivotal role in facilitating the translocation of T4 from the bloodstream into the CSF across the blood-CSF barrier, particularly during phases of accelerated cerebral development [29]. Thyroid hormones also enter the brain directly via the blood–brain barrier through specific transporters [30]. Additionally, TTR complexes with RBP to prevent renal filtration, protecting vitamin A reserves from excretion and ensuring the bioavailability of retinol for visual and immune functions [31].

TTR also acts as a molecular chaperone that maintains cellular homeostasis by ensuring proper protein folding and preventing aggregation [32]. In environments such as plasma and CSF, it inhibits the formation of amyloid fibrils by interacting with intermediates of proteins, including Aβ and islet amyloid polypeptide (IAPP) [33]. By binding to Aβ peptides, TTR reduces their cytotoxicity and suppresses plaque formation [34]. Furthermore, TTR promotes neurite outgrowth and neuronal survival [35] and modulates signaling pathways integral to synaptic plasticity and memory formation [36]. It is also expressed in the peripheral nervous system, particularly in Schwann cells [37]. Post-injury, elevated TTR promotes Schwann cell migration and regulates the extracellular matrix to support nerve regeneration.

The functional TTR tetramer is essential for cardiac health. Research indicates that lower TTR concentrations reflect tetramer instability and an increased risk of incident HF [38]. Concentrations below 20 mg/dL are associated with higher all-cause and cardiovascular mortality compared with levels between 20 and 40 mg/dL [39].

### 2.3. TTR Gene

**Figure 3:**
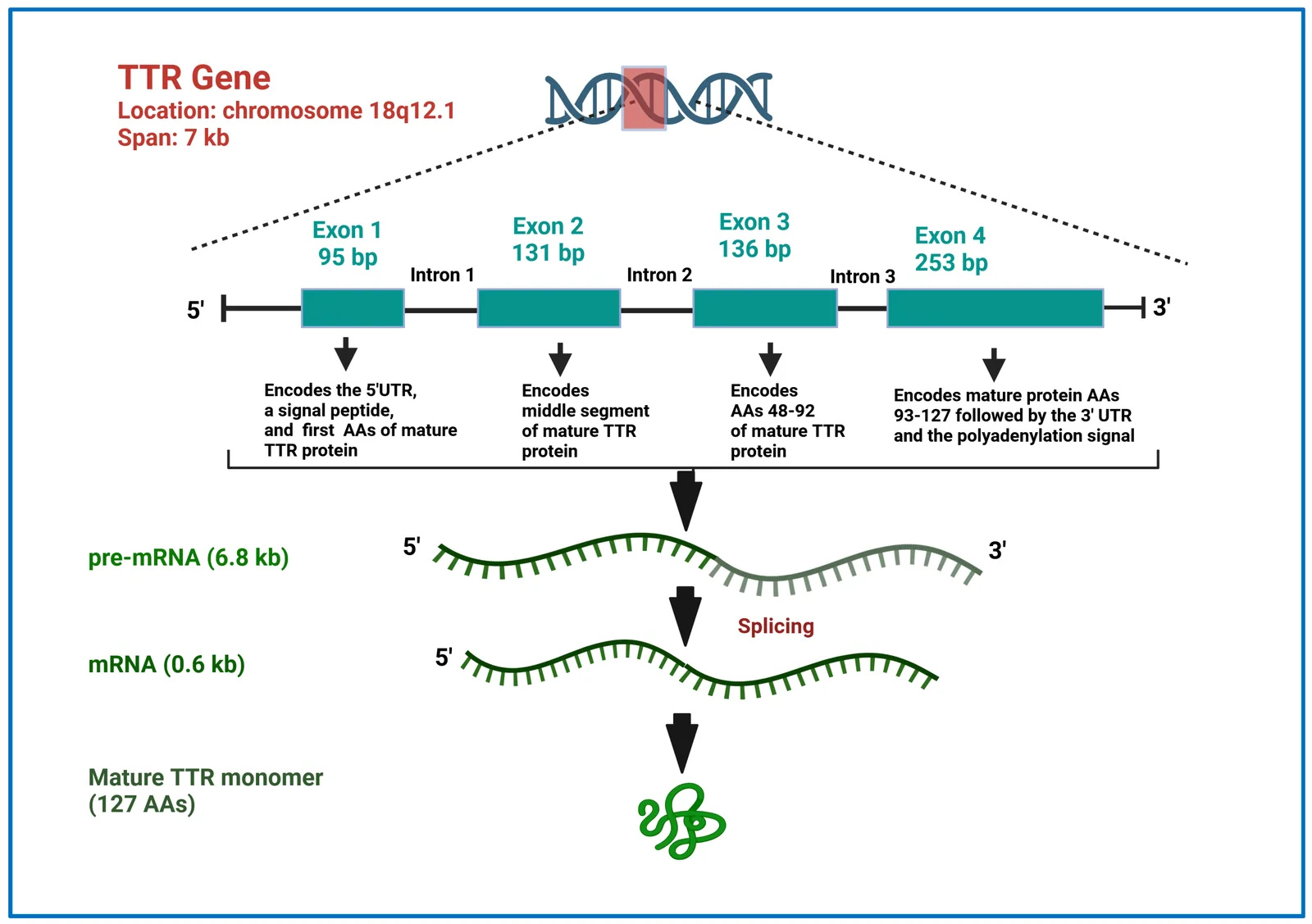
Genomic organization and transcriptional pathway of the human transthyretin (TTR) gene. Schematic representation of the 7 kb TTR locus situated on chromosome 18q12.1. The gene consists of four distinct exons separated by three introns: Exon 1 (95 bp) encodes the 5′ untranslated region (UTR), a signal peptide, and the initial amino acids of the mature protein; Exon 2 (131 bp) and Exon 3 (136 bp) encode intermediate segments; Exon 4 (253 bp) encodes the terminal amino acids, the 3′ UTR, and the polyadenylation signal. Transcription yields a 6.8 kb pre-mRNA transcript, which undergoes splicing to generate a stable 0.6 kb mature mRNA, ultimately translating into the functional 127-amino-acid mature TTR monomer. Created in BioRender. Triposkiadis, F. (2026) https://BioRender.com/82qm351.

The TTR gene, located on chromosome 18 (18q12.1), spans approximately 7 kb and comprises four exons and three introns [40,41]. Its structure includes TATA box-like and CAAT box-like sequences [42] (Figure 3). The TATA box-like sequence defines the transcription initiation site, while the upstream CAAT box-like sequence enhances transcription rates. Together, these elements facilitate efficient gene expression [42]. TTR mRNA is translated into pro-TTR monomers, which are cleaved into native monomers featuring an N-terminal region essential for tetramer assembly [18]. TTR expression in the CP enables protein secretion into the CSF through regulatory mechanisms distinct from hepatic synthesis [43], involving different tissue-specific nuclear factors despite high expression in both sites [44].

Over 200 TTR sequence variants exist. Most disease-associated changes are single-nucleotide substitutions, though non-amyloidogenic and structural variants occur. Reported totals vary due to differing database criteria; here, “pathogenic variant” refers specifically to those causing ATTR [45]. Although some variants are well characterized, biochemical and clinical data for rare mutations remain limited. While amino acid substitutions predominate, structural deletions and duplications also occur [45,46].

#### 2.3.1. Exon 1

Exon 1 of the TTR gene encodes the 5′-UTR, a signal peptide, and the start of the mature protein. Unlike common mutations in exons 2–4 that destabilize the secreted tetramer into amyloidogenic monomers [18,45], exon 1 mutations primarily disrupt the secretory pathway via the signal peptide. Consequently, because exon 1 focuses on secretion rather than protein stability, these mutations are rare and differ mechanistically from classic amyloidogenic phenotypes [47].

#### 2.3.2. Exon 2

Exon 2 of the TTR gene encodes amino acids 4–47, forming a critical part of the monomer’s core. This segment includes β-strands A, B, and C, and the AB loop, contributing to the T4 binding channel [18,45]. As a primary focus of ATTRv research, exon 2 contains several mutation hotspots. Identified pathogenic mutations include: (a) *Val30Met* (*p.Val50Met*), the classic variant underlying familial amyloid polyneuropathy (FAP) [48]; (b) *Val20Ile* (*p.Val40Ile*): Mainly causes late-onset, severe ATTRv-CM, resembling wild-type ATTR but typically appearing in the 60s or 70s [49]; (c) *Cys10Arg* (*p.Cys30Arg*): A rare, aggressive variant causing a mixed phenotype of rapid polyneuropathy (PN) and vitreous amyloid opacities [50]; (d) *Ala45Val* (*p.Ala65Val*) and *Ala45Asp* (*p.Ala65Asp*), typically manifesting as late-onset mixed cardiac and neurological phenotypes, including severe orthostatic hypotension and restrictive cardiomyopathy [51]; (e) *Asp18Asn/*(*p.Asp38Asn*), a cardiac-predominant variant with minimal/absent neuropathy and autonomic involvement [52].

#### 2.3.3. Exon 3

Exon 3 of the TTR gene encodes amino acids 48–92, forming critical structural components of the monomer: β-strands D, E, and F, and the surface-exposed EF helix and loop [18,45]. This region mediates interactions with RBP for vitamin A transport. Clinically, exon 3 is a hotspot for pathogenic mutations associated with ATTRv, including variants causing familial amyloid cardiomyopathy and severe neurological dysfunction, including: (a) *Glu89Gln* (*p.Glu109Gln*), a highly cardiotoxic variant characterized by aggressive and rapidly progressive ATTRv-CM [53]; (b) *Phe64Leu* (*p.Phe84Leu*), a variant typically presenting as late-onset, slowly progressive sensorimotor polyneuropathy, predominantly observed in cohorts of Italian and American descent [54]; and (c) *severe leptomeningeal variants, such as Tyr69His* (*p.Tyr89His*) *and Gly53Arg* (*p.Gly73Arg*), which lead to rare and highly specialized oculoleptomeningeal amyloidosis [55]. Exon 3 maintains the protein’s structural integrity; therefore, mutations in this region often result in the most kinetically or thermodynamically unstable TTR variants observed clinically.

#### 2.3.4. Exon 4

TTR gene exon 4 encodes amino acids 93–127 and the 3′-UTR, forming structural components essential for protein assembly and T4 binding [18,45]. Mutations in this region disrupt tetramer stability, causing various ATTRv phenotypes: (a) *Val122Ile* (*p.Val142Ile*), common in African populations, leads to late-onset ATTRv-CM [56,57]; (b) *Ile107Val* (*p.Ile127Val*), prevalent in Greek and Sicilian populations, presents as a mixed phenotype of polyneuropathy and cardiomyopathy [58]; (c) *Glu122Lys* (*p.Glu142Lys*) variant has been reported in patients with ATTR-CM in a recent single-center study conducted in southern Poland [59]; and (d) *Tyr114Cys* (*p.Tyr134Cys*) is associated with oculoleptomeningeal and peripheral involvement [60,61]. Since exon 4 encodes the C-terminal lock required for tetrameric stability, variations in this domain directly drive many prevalent cardiac phenotypes.

## 3. TTR Amyloidogenesis

**Figure 4:**
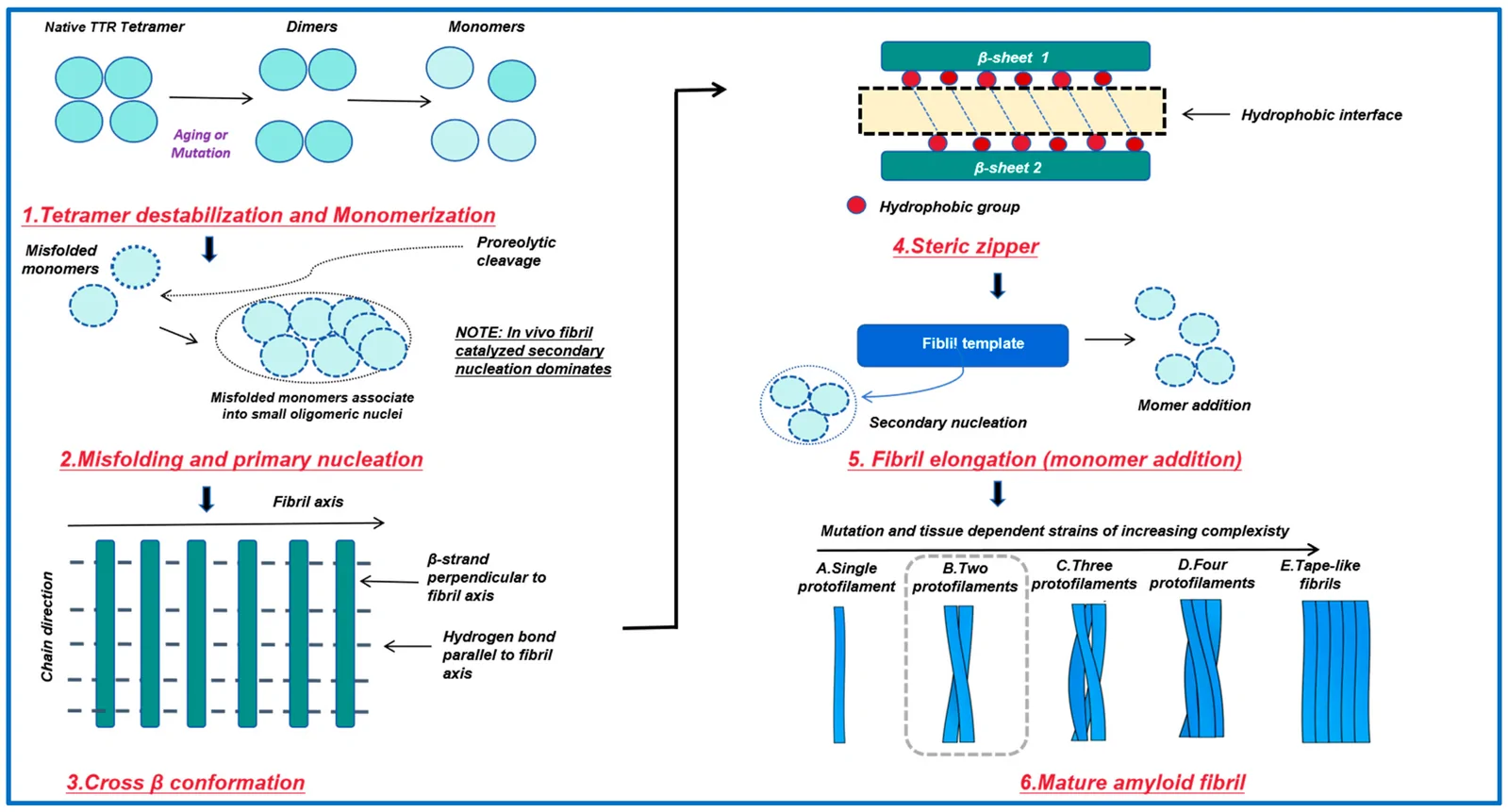
The mechanistic pathway of transthyretin (TTR) amyloidogenesis involves several key stages. The process begins with tetramer dissociation into unstable monomers, followed by misfolding and primary nucleation into oligomeric nuclei. The assembly forms a cross-β core, stabilized by a steric zipper mechanism that excludes water at the β-sheet interface. Existing fibrils promote further amyloid accumulation through two distinct processes: endwise monomer addition drives fibril elongation, whereas fibril-surface-catalyzed secondary nucleation generates new aggregation-competent nuclei. Finally, mature amyloid fibrils form, exhibiting structural polymorphism ranging from single protofilaments to complex twisted hierarchies and ribbon-like structures, influenced by mutations and the microenvironment. Created in BioRender. Triposkiadis, F. (2026) https://BioRender.com/88rpqum.

TTR tetramer dissociation and conformational transition lead to insoluble amyloid fibril formation (amyloidogenesis) [62]. This process, driven by TTR mutations in ATTRv or wild-type misfolding in ATTRwt, may be divided into the following stages (Figure 4).

### 3.1. Protein Destabilization and Monomerization

The rate-limiting step in TTR amyloidogenesis is the destabilization and dissociation of the tetramer into monomers [63]. This process often involves a dimeric intermediate before reaching the monomeric state [64]. Following dissociation, the liberated monomers undergo conformational transitions into amyloidogenic states [65]. Misfolded monomers expose hydrophobic regions and β-strands, promoting self-association. 

ATTRv results from TTR gene mutations that destabilize the native tetramer, thereby promoting protein dissociation and subsequent amyloid fibril formation [66]. This instability facilitates systemic deposition and the creation of toxic oligomeric intermediates, which cause proteotoxicity by damaging cell membranes and signaling pathways [67]. In ATTRwt, TTR structural stability declines with age despite a normal sequence [68]. Complex mechanisms, including conformational changes [69] and oxidative stress [70], destabilize the protein tetramer. Low pH promotes aggregation by triggering tetramer dissociation and monomer misfolding [71]. Even at neutral pH, physical stressors such as agitation or turbulent flow can induce TTR aggregation and fibril formation by driving conformational changes.

Beyond tetramer dissociation, proteolysis also facilitates TTR amyloid formation [72]. Specific fragments, such as C-terminal 49–127, occur in ex vivo deposits and subsequently co-aggregate with full-length TTR to accelerate amyloid formation.

### 3.2. Misfolding and Primary Nucleation

Following tetramer dissociation, TTR monomers undergo structural reorganization into unstable, non-native intermediates [73]. These destabilized monomers aggregate into soluble oligomers, which eventually form a stable nucleus. This process triggers rapid polymerization, leading to the deposition of insoluble amyloid fibrils in tissues and organs [73].

Mechanistically, monomer misfolding involves partial, rather than global, unfolding: the CD loop and the edge β-strands (particularly strand C and the adjacent AB loop) detach first, exposing previously buried hydrophobic surfaces that are shielded from solvent in the native tetramer, while the core β-sandwich retains substantial native-like structure in the earliest intermediates [65,74]. Self-association of these partially unfolded monomers is thermodynamically distinct from simple aggregation: proximity of exposed hydrophobic patches nucleates a small oligomeric assembly whose conformation is not yet the final cross-β fold, but which lowers the energetic barrier for recruitment of additional monomers (primary nucleation). Only after this initial nucleus forms do the constituent monomers undergo the previously described more extensive conformational rearrangement, which converts the native β-sandwich into the amyloid cross-β architecture; thus, misfolding and nucleation are sequential but mechanistically coupled steps, and the soluble oligomeric and other nonfibrillar TTR species are considered important mediators of proteotoxicity in ATTR amyloidogenesis.

### 3.3. Cross-β Conformation

The cross-β conformation is the core structural motif of amyloid fibrils, characterized by β-strands oriented perpendicularly to the fibril axis [75]. These strands form stable, continuous β-sheets linked by hydrogen bonds. While the exact conversion mechanism is unclear, amyloid formation involves partial protein unfolding to allow polypeptide chains to reassemble into the cross-β structure [76].

**Figure 5:**
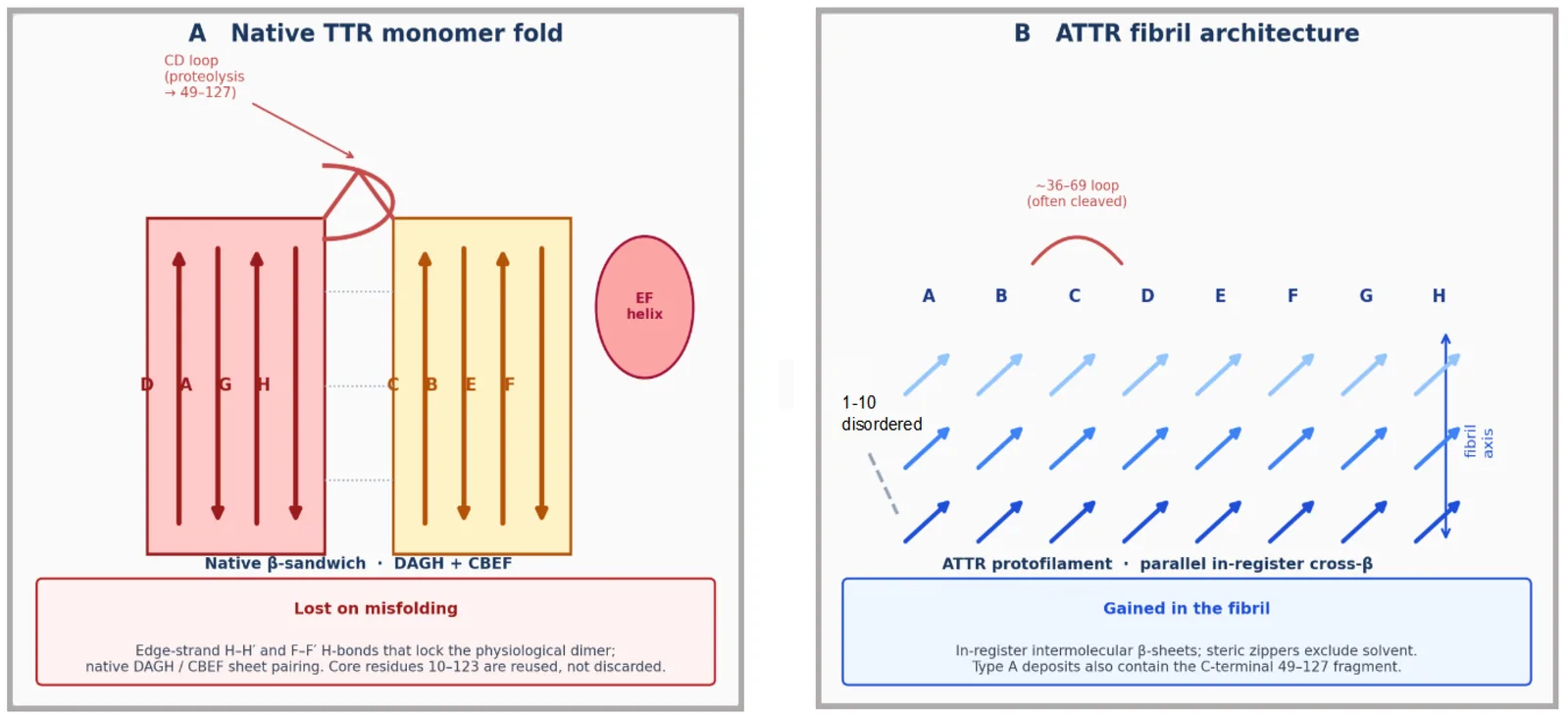
Native transthyretin (TTR) β-sandwich versus the TTR amyloidosis (ATTR) fibril fold. (**A**) Native monomer fold. The 127-residue subunit is a β-sandwich of two four-stranded sheets, DAGH and CBEF, with a solvent-exposed EF helix–loop that forms the RBP interface. Monomer misfolding is partial rather than global: the CD loop and edge strand C (with the adjacent AB loop) detach first, exposing hydrophobic surfaces that are buried in the tetramer, while the core sandwich retains substantial native-like structure in the earliest intermediates. Proteolysis of the CD loop generates the C-terminal 49–127 fragment enriched in cardiac Type A deposits. Native inter-strand contacts lost on misfolding include the edge-strand H–H′ and F–F′ hydrogen bonds that stabilize the physiological dimer, together with the DAGH/CBEF sheet pairing. (**B**) ATTR protofilament. High-resolution analyses show that the amyloid core spans approximately residues 10–123 of the native chain, connected by a disordered loop. The same eight strands (A–H) are reused but reorganized into a parallel, in-register intermolecular cross-β architecture. Residues 1–10 are disordered; the ~36–69 connecting segment is frequently disordered or removed by proteolysis. Mature pathogenic fibrils adopt the parallel in-register geometry, despite the greater intrinsic stability of isolated antiparallel sheets, because in-register packing supports continuous hydrophobic ladders and high-complementarity steric zippers that exclude solvent. Type A deposits contain both fragmented and full-length TTR; Type B deposits contain full-length protein only. CD loop, the loop between β-strands C and D. Created in BioRender. Triposkiadis, F. (2026) https://BioRender.com/8827yvk.

Empirical data show that the spatial arrangement of β-strands in the amyloid core differs significantly from that of the native homotetramer [77]. High-resolution analyses confirm that the amyloid core consists of two peptide segments, spanning approximately residues 10–123 of the native 127-residue polypeptide, connected by a disordered loop. This core sequence subsumes most of the native eight-stranded β-sandwich (strands A–H) but is reorganized into a cross-β architecture, so that the native inter-strand contacts (including the edge-strand H–H′ and F–F′ hydrogen bonds that stabilize the physiological dimer) are lost and replaced by new, in-register inter-molecular β-sheet contacts between stacked monomers [78] (Figure 5).

Within the cross-β architecture, β-strands are organized into parallel or antiparallel geometries. Parallel strands align in the same direction, while antiparallel strands are oriented opposite. These orientations dictate distinct hydrogen-bonding patterns [79]: parallel alignment results in skewed hydrogen bonds because the C=O and N-H groups are not directly aligned. These bonds are distributed evenly but are less energetically optimal than linear ones. In contrast, antiparallel alignment allows for straight hydrogen bonds with alternating spacing. While isolated antiparallel β-sheets are more thermodynamically stable, mature pathogenic amyloid fibrils typically adopt a parallel, in-register architecture [80]. This preference is driven by two main factors: in-register alignment enables continuous hydrophobic interactions or charge ladders along the fibril axis, while parallel alignment creates high-complementarity steric zippers that exclude solvent. These interactions compensate for the suboptimal geometry of parallel hydrogen bonds [66].

### 3.4. Steric Zipper Formation

Cryo-electron microscopy (cryo-EM) of TTR fibrils has confirmed a dense core structure composed of parallel β-sheets from misfolded TTR proteins [81]. Within this structure, amino acid side chains from the TTR protein interlock via a “steric zipper” motif. This configuration excludes water from the hydrophobic interior, creating a resilient, insoluble architecture resistant to endogenous proteolytic clearance and enzymatic breakdown [82].

### 3.5. Fibril Elongation

TTR fibril ends serve as specific templates for growth [83]. Circulating monomers are recruited to these sites, where they undergo conformational changes to integrate into the existing fibril structure [84,85]. This recruitment and structural adaptation drive the continuous longitudinal expansion of TTR protofilaments.

### 3.6. The Mature Amyloid Fibril

In ATTR amyloidosis, protofilaments are intermediate structures formed by TTR aggregation that serve as the building blocks for mature amyloid fibrils [81,86]. Composed of a cross-β sheet architecture, several protofilaments intertwine to form fibrils that deposit in organs such as the heart and peripheral nerves [87]. These deposits are categorized into Type A and Type B [81]: Type A fibrils contain both fragmented and full-length TTR, while Type B consists solely of full-length protein [77,88]. Type A deposits are more prevalent in cardiac cases, where C-terminal fragments may accelerate fibril stabilization [89]. Conversely, Type B deposits often characterize systemic cases with limited cardiac burden, highlighting how site-specific aggregation kinetics influence disease severity [90]. Additionally, non-coding variants, fibril composition, and epigenetic factors contribute to phenotypic diversity and clinical progression of ATTRv [5].

After deposition, amyloid fibrils cause damage through several mechanisms. These include mechanical stiffening of tissues, leading to restrictive cardiomyopathy [91] and compressive neuropathy [92]. Non-fibrillar species further induce cellular injury via membrane disruption and oxidative stress [93]. Additionally, mitochondrial and endoplasmic reticulum stress [94] trigger inflammation [95] and microvascular dysfunction [96]. These processes manifest systemically, severely affecting the nervous system and gastrointestinal tract [6].

## 4. Experimental Models of ATTR

Animal and cellular models have been fundamental in elucidating TTR amyloidogenesis and facilitating the preclinical evaluation of stabilizers and gene-silencing strategies [97]. Early transgenic murine models expressing human TTR variants, such as Val30Met, exhibited age-dependent systemic deposits within the gastrointestinal tract, cardiovascular system, and kidneys; however, they generally failed to replicate the peripheral and autonomic neuropathy central to human ATTRv-PN. Transgenic mice expressing wild-type human TTR have demonstrated cardiac and renal TTR deposition with senescence, providing a model for aspects of ATTRwt. A significant experimental constraint arises from the fact that endogenous murine TTR can form relatively stable heterotetramers with human TTR, thereby reducing amyloidogenicity. Consequently, certain models utilize a murine TTR-null background or additional proteostasis perturbations to enhance deposition [98]. More recent humanized knock-in models, which express amyloidogenic human TTR alleles from the endogenous locus, more accurately reproduce nerve deposition, sensory abnormalities, and early neuropathic phenotypes [99]. Additional biological systems—including *Drosophila, Caenorhabditis elegans*, and patient-derived induced pluripotent stem cells—enable rapid mechanistic and therapeutic investigations [100,101]. Nevertheless, because no single model fully recapitulates the complete human spectrum of cardiac, neurological, ocular, and leptomeningeal ATTR, experimental findings must be interpreted across complementary model systems.

## 5. Histological and Fluorescent Detection of Amyloid

Definitive diagnosis of tissue ATTR deposition relies on histochemical staining, since all amyloid types share a common cross-β fibrillar architecture that produces a characteristic tinctorial pattern regardless of the precursor protein. Congo red remains the diagnostic gold standard, producing apple-green birefringence under cross-polarized light due to the ordered, crystalline-like stacking of dye molecules along the fibril axis; alkaline Congo red protocols and confirmatory restaining improve specificity when initial results are equivocal [102]. Thioflavin T and Thioflavin S offer greater sensitivity via fluorescence emission upon binding β-sheet-rich structures but are less specific than Congo red, since they can also bind non-amyloid β-sheet-containing structures, and dye-binding kinetics can be confounded by co-administered small-molecule TTR stabilizers or other exogenous compounds that compete for the same binding grooves [103,104]. Once amyloid is confirmed histologically, immunohistochemistry or mass spectrometry-based proteomics is required to subtype the fibril protein (e.g., distinguishing ATTR from AL or other amyloidoses), as tinctorial stains alone cannot identify the causative precursor. Newer fluorescent and near-infrared probes, developed primarily for amyloid-β imaging in Alzheimer’s disease, are also being explored for noninvasive, quantitative amyloid imaging that could eventually complement or reduce reliance on invasive tissue biopsy in systemic amyloidoses [103].

## 6. Treatment

**Figure 6:**
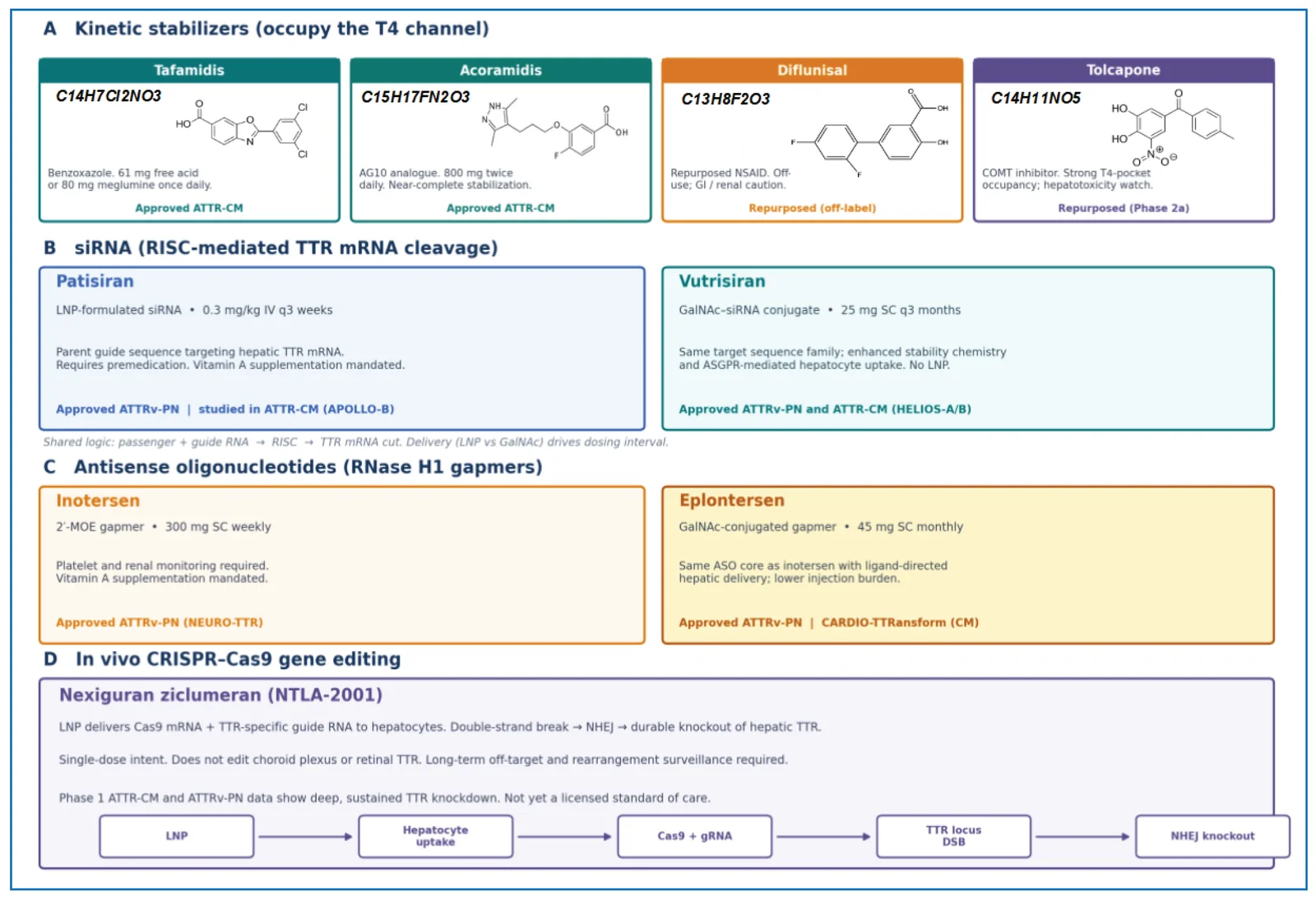
Chemical and modular structures of transthyretin (TTR) amyloidosis (ATTR) disease-modifying therapies. Therapeutic classes mapped onto discrete steps of the pathogenic cascade. (**A**) Kinetic stabilizers occupy the thyroxine (T4) channel and slow tetramer dissociation without eliminating circulating TTR or retinol-binding protein (RBP) transport. Tafamidis (61 mg free acid or 80 mg meglumine once daily) and acoramidis (800 mg twice daily). Diflunisal is a repurposed nonsteroidal anti-inflammatory drug (NSAID) used off-label. Tolcapone, a catechol-O-methyltransferase (COMT) inhibitor approved for Parkinson disease, binds the T4 pocket of wild-type and V122I TTR more tightly than tafamidis in structural studies and showed dose-dependent tetramer stabilization in a phase 2a proof-of-concept study; hepatotoxicity remains a development constraint, and the agent is not approved for ATTR. (**B**) siRNA therapeutics recruit RNA-induced silencing complex (RISC) to cleave hepatic TTR mRNA. Patisiran is a lipid nanoparticle (LNP)-formulated duplex given intravenously every 3 weeks. Vutrisiran is a N-acetylgalactosamine (GalNAc)–siRNA conjugate given subcutaneously every 3 months; asialoglycoprotein receptor (ASGPR)-mediated hepatocyte uptake accounts for the longer dosing interval. Both require vitamin A supplementation. (**C**) Antisense oligonucleotides (ASO) act as RNase H1-recruiting gapmers. Inotersen (weekly subcutaneous) and eplontersen (GalNAc-conjugated, monthly subcutaneous) share an ASO core; ligand-directed delivery reduces injection burden for eplontersen. Platelet and renal monitoring applies particularly to inotersen. (**D**) In vivo clustered regularly interspaced short palindromic repeats (CRISPR) and CRISPR-associated protein 9 (Cas9) (CRISPR-Cas9) editing (nexiguran ziclumeran, NTLA-2001) delivers Cas9 mRNA and a TTR-specific guide RNA to hepatocytes in a lipid nanoparticle. A double-strand break (DSB) resolved by non-homologous end joining produces durable hepatic knockout after a single dose. Extrahepatic synthesis (choroid plexus, retina) is not edited, and long-term surveillance for off-target cleavage and rearrangements is required. Stabilizers and silencers reduce new precursor supply but leave existing fibrils in place; none of the agents in this figure is an amyloid-depleting antibody. ATTR-CM, ATTR cardiomyopathy; ATTRv-PN, hereditary ATTR polyneuropathy; gRNA, guide RNA; NHEJ, non-homologous end joining. Created in BioRender. Triposkiadis, F. (2026) https://BioRender.com/k0qlj4g.

Therapeutic strategies for ATTR amyloidosis target discrete steps of the pathogenic cascade. Kinetic stabilizers occupy the T4 channel and slow tetramer dissociation; siRNA and antisense oligonucleotides suppress hepatic TTR synthesis; CRISPR-Cas9 editing aims for durable hepatic knockout; and amyloid-depleting monoclonal antibodies are intended to clear established deposits (Figure 6). These mechanisms are complementary rather than interchangeable. Stabilizers and silencers reduce new precursor supply but leave existing fibrils in place; depleting antibodies address tissue burden but do not stop ongoing production. Combination or sequential regimens are therefore biologically rational—particularly stabilizer or silencer plus an anti-fibril antibody—but remain incompletely tested for additive efficacy, overlapping toxicity, and extrahepatic residual disease (choroid plexus, retina). Early diagnosis and a multidisciplinary pathway remain essential, because no current regimen reverses advanced organ replacement.

## 7. TTR Stabilizers

**Figure 7:**
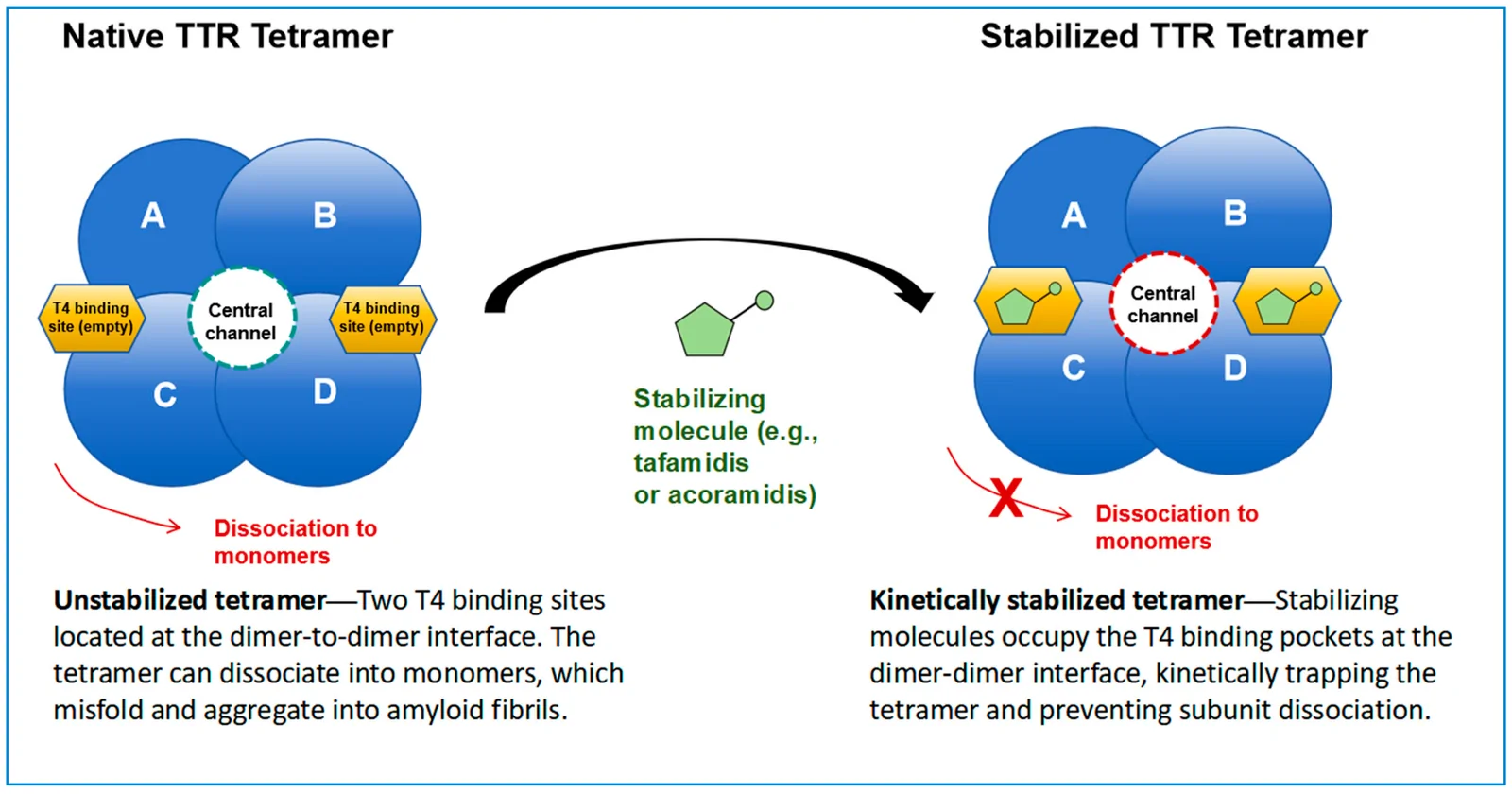
Schematic comparing unbound versus drug-bound TTR tetramers. In the unstabilized pathway (**left**), the native tetramer dissociates into dimers and monomers; these misfolded monomers then assemble into amyloid fibrils, driving ATTR pathology. In the stabilized pathway (**right**), ligands bind the central channel to stabilize the tetramer, preventing dissociation and fibril formation. Some ligands exhibit negative cooperativity. Overall, stabilization reduces dissociation rates and downstream amyloidogenesis. TTR, transthyretin; M, monomer; T4, thyroxine; ATTR, transthyretin amyloidosis. Created in BioRender. Triposkiadis, F. (2026) https://BioRender.com/wajwmi4.

TTR stabilizers bind to and stabilize the TTR protein tetramer to prevent amyloidogenesis. By occupying T4-binding sites, they inhibit tetramer dissociation, the rate-limiting step in fibril formation, and mitigate amyloidosis across wild-type and genetic variants (Figure 7) [105]. Among the stabilizers developed, tafamidis and acoramidis are the only ones currently approved by major regulatory agencies.

### 7.1. Tafamidis

Tafamidis is a selective oral TTR stabilizer for ATTR-CM. It binds to TTR thyroxine sites, stabilizing the protein’s native structure [106]. The pivotal ATTR-ACT (Transthyretin Amyloid Cardiomyopathy Clinical Trial) demonstrated that tafamidis significantly reduces all-cause mortality and cardiovascular hospitalizations compared with placebo over 30 months [107]. Subsequent long-term extension data confirmed the survival and functional benefit [108]. Additionally, tafamidis slowed eGFR decline and improved chronic kidney disease staging, maintaining a safety profile similar to placebo [109]. ATTR-ACT analysis showed that tafamidis slowed deterioration in four echocardiographic parameters compared with placebo [110]. The tafamidis oral dose in ATTR-CM is 80 mg with the meglumine formulation (4 × 20 mg capsules qd) or 61 mg with the bioequivalent free-acid formulation (1 capsule qd).

In the 18-month Fx-005 study of adults with early-stage V30M ATTRv-PN, tafamidis did not show statistically significant differences from placebo in the coprimary endpoints (NIS-LL [Neuropathy Impairment Score in the Lower Limbs] response rate and change in Norfolk QOL-DN [Norfolk Quality of Life-Diabetic Neuropathy] scores) [111]. However, per-protocol analyses showed significantly better outcomes for tafamidis, with 98% of patients achieving TTR stabilization [111]. Subsequently, the open-label extension (Fx-006) showed that tafamidis maintained neurological function and QOL over 30 months, with placebo-to-tafamidis transitioners showing a significantly slower decline [112]. Regulatory approval of tafamidis for ATTRv-PN varies by jurisdiction; it is not approved for this indication in the United States.

THAOS (Transthyretin Amyloidosis Outcomes Survey) real-world evidence shows that tafamidis significantly improves survival rates in ATTR-CM patients at 30 and 42 months compared with untreated controls [113]. Similarly, an analysis of 339 THAOS patients with a mixed phenotype confirmed that tafamidis significantly improved 30-month survival and reduced annual cardiovascular hospitalizations compared with untreated patients [114].

Tafamidis is well tolerated. In ATTR-CM, it significantly lowers mortality and hospitalization rates. Clinical monitoring is advised for patients with hepatic or renal impairment, those using CYP2C9 substrates, or during pregnancy.

### 7.2. Acoramidis

Acoramidis is a TTR kinetic stabilizer that demonstrated stabilization of variant and wild-type TTR in preclinical human serum evaluations [115]. Acoramidis is indicated for ATTR-CM in adults based on the Phase 3 ATTRibute-CM study. Over 30 months, acoramidis 800 mg twice daily significantly outperformed placebo across key primary outcomes, including mortality, hospitalizations, and functional measures [116]. It reduced mortality and cardiovascular hospitalizations while preserving functional capacity and quality of life versus placebo. The beneficial effect of acoramidis was observed consistently in the ATTRwt-CM and ATTRv-CM groups [117].

Adverse event and discontinuation rates were similar overall between groups, but acoramidis was associated with more frequent gastrointestinal adverse events than placebo (diarrhea, 11.6% vs. 7.6%; upper abdominal pain, 5.5% vs. 1.4%) and an early, reversible decline in eGFR that subsequently stabilized [118]. However, acoramidis reduced mortality and cardiovascular hospitalizations by month 42, with continuous treatment outperforming a delayed start from placebo [118]. In the ATTRibute-CM trial, acoramidis increased serum TTR (sTTR) levels, which partially mediated a lower risk of cardiovascular death and hospitalization. This suggests that sTTR may serve as a clinically informative biomarker after stabilizer initiation [119]. Data on acoramidis’s efficacy and safety in ATTR-PN patients is unavailable. The Phase 3 ACT-EARLY trial (NCT06563895) is currently evaluating acoramidis for preventing transthyretin amyloidosis in asymptomatic gene carriers.

### 7.3. Diflunisal

Clinical trials, including a 2-year randomized study in ATTRv-PN patients, showed that diflunisal (250 mg twice daily) slows disease progression and preserves quality of life [120]. However, diflunisal is a weaker stabilizer than tafamidis or acoramidis, and it is not currently approved for use.

### 7.4. Tolcapone

Tolcapone, a catechol-O-methyltransferase inhibitor approved for Parkinson’s disease, has been repurposed as a candidate TTR kinetic stabilizer [121]. Tolcapone binds specifically to TTR in human plasma, stabilizes the native tetramer in vivo in mice and humans, and inhibits TTR-associated cytotoxicity; crystal structures show it docks into the T4 pocket of both wild-type and V122I-variant TTR more effectively than tafamidis [121]. A phase 2a proof-of-concept study in hereditary ATTR patients, asymptomatic carriers, and healthy volunteers confirmed dose-dependent TTR stabilization with no serious adverse events, supporting further clinical development [122]. Several tolcapone derivatives designed to reduce its hepatotoxicity liability while preserving TTR-stabilizing activity are in preclinical development, representing a promising drug-repurposing avenue for TTR amyloidosis [123].

## 8. Short Interfering RNAs (siRNAs)

**Figure 8:**
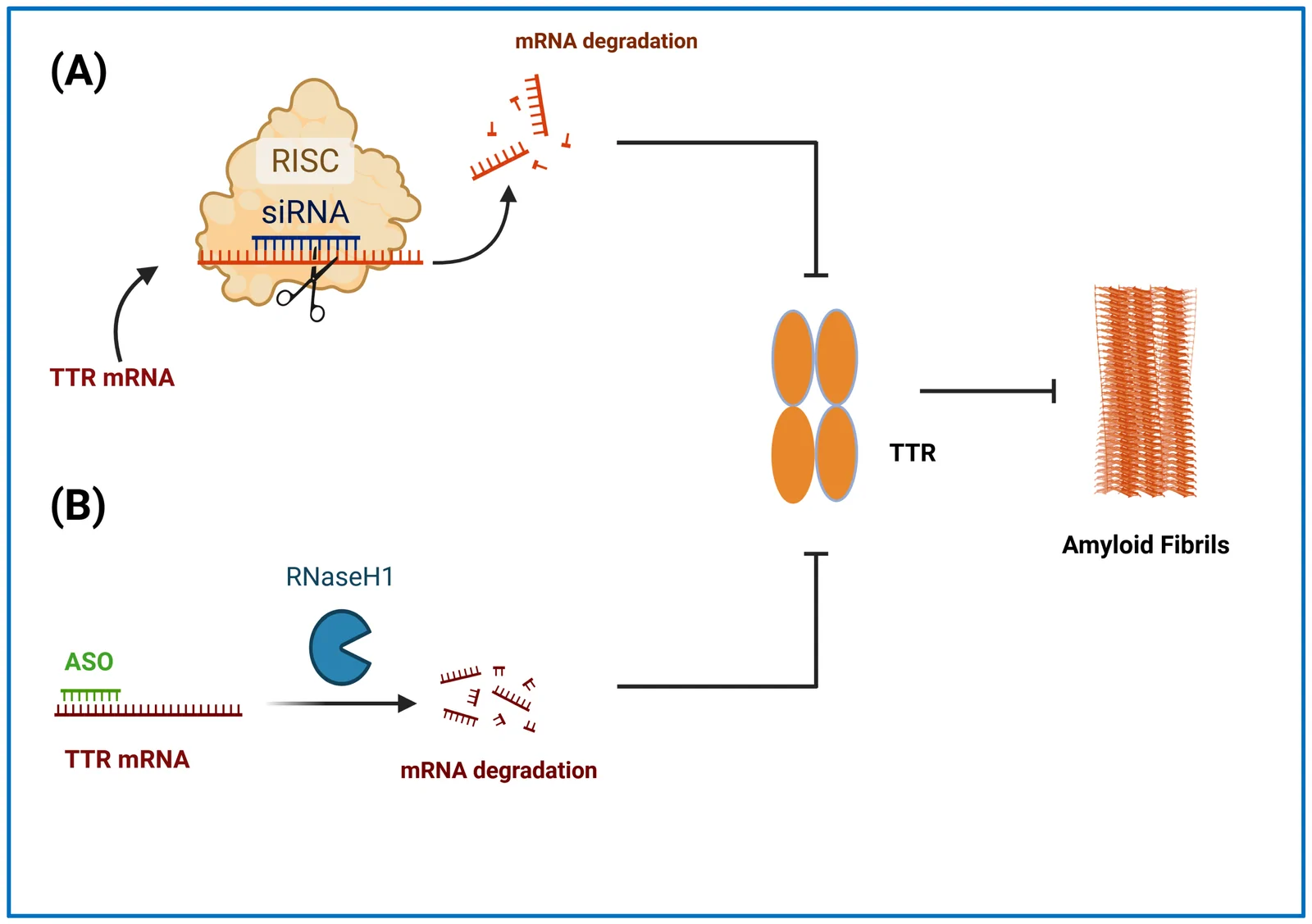
Comparative mechanism of TTR gene-silencing therapeutics. (**A**) siRNA pathway: double-stranded siRNA enters the cytoplasm and joins the RNA-induced silencing complex (RISC) and subsequently cleaves the target TTR mRNA. (**B**) ASO pathway: single-stranded ASOs bind to TTR mRNA, forming a heteroduplex. This structure triggers RNase H1 to hydrolyze the RNA strand. Both modalities achieve targeted TTR mRNA degradation, resulting in decreased circulating TTR protein and reduced amyloid deposition in tissues. TTR, transthyretin; siRNA, short interfering RNA; ASO, antisense oligonucleotide. Created in BioRender. Triposkiadis, F. (2026) https://BioRender.com/svwtiia.

siRNAs utilize the RNA-induced silencing complex to degrade specific transcripts (Figure 8).

### 8.1. Patisiran

Patisiran is the first siRNA therapeutic approved for ATTRv-PN [124]. It contains a modified, double-stranded siRNA targeting TTR mRNA, delivered via lipid nanoparticles (LNPs) for hepatocyte uptake following IV administration [125]. The pivotal APOLLO (A Placebo-controlled, Open-label, Long-term Objective study of the drug Patisiran) trial randomized 225 patients with ATTRv-PN to receive either patisiran (0.3 mg/kg IV every 3 weeks) or placebo [126]. At 18 months, patisiran treatment resulted in a mean 6.0-point improvement in the modified Neuropathy Impairment Score +7 (mNIS+7), compared with deterioration in the placebo group, along with significant improvements in the Norfolk QOL-DN score [126]. Serum TTR levels decreased by 81% from baseline. Open-label extension data showed sustained efficacy over five years, and a cardiac substudy reported improvements in biomarkers, structure, and functional capacity in cardiomyopathy patients [127]. These results informed the subsequent APOLLO-B trial, in which patisiran demonstrated beneficial effects on functional capacity and cardiac biomarkers in patients with ATTR-CM [128]. However, it is important to note that the FDA issued a complete response letter in October 2023, declining approval for this specific indication.

To reduce infusion reaction risks, premedicate patients with an intravenous corticosteroid, oral acetaminophen, and H1/H2 blockers at least 60 min before each dose [129]. Daily vitamin A supplementation (~2500 IU) is recommended since TTR carries RBP. Common side effects include infusion reactions and peripheral edema. Patisiran may cause vitamin A deficiency and is not recommended during pregnancy.

### 8.2. Vutrisiran

Vutrisiran is a second-generation siRNA therapeutic indicated for the treatment of both ATTRv-PN [130] and ATTR-CM [131]. Although vutrisiran targets the identical TTR sequence as patisiran, its conjugation to an N-acetylgalactosamine (GalNAc) ligand facilitates specific binding to the asialoglycoprotein receptor, which is predominantly expressed on hepatocytes [132]. This mechanism enables rapid vutrisiran endocytosis and endosomal escape, delivering sufficient siRNA to the cytoplasm for robust RNAi responses in vivo. Furthermore, GalNAc conjugation allows for effective vutrisiran levels via subcutaneous administration.

In the HELIOS-A trial, a randomized open-label study of 164 patients with ATTRv-PN, vutrisiran (25 mg SC quarterly) was compared with the external APOLLO placebo. Vutrisiran reduced serum TTR levels by 81–88% from baseline, showed significant improvements in mNIS+7, and demonstrated noninferiority to patisiran [133]. HELIOS-B trial results showed that adding the treatment to standard care significantly reduced mortality and cardiovascular hospitalizations in ATTR-CM patients compared with placebo [134,135].

GalNAc conjugation eliminates the need for LNP delivery and associated premedication. Injection-site reactions are typically mild. Daily vitamin A supplementation (2500 IU) remains recommended, consistent with patisiran protocols. Common adverse events include mild injection-site reactions and arthralgia [136]. Vutrisiran’s quarterly SC dosing offers greater convenience than patisiran while maintaining comparable efficacy.

## 9. Antisense Oligonucleotides (ASOs)

Antisense oligonucleotides (ASOs), such as inotersen and eplontersen, alleviate amyloid transthyretin (ATTR) amyloidosis by inhibiting hepatic TTR protein synthesis (Figure 8). This mechanism involves binding to and degrading TTR mRNA, thereby precluding the production of misfolded proteins [137]. ASOs inhibit TTR synthesis to lower circulating protein levels, slowing amyloid formation and stabilizing disease progression in patients with polyneuropathy and cardiomyopathy.

### 9.1. Inotersen

This ASO therapy is designed to address ATTRv by inhibiting TTR production. By inducing the enzymatic degradation of TTR mRNA, the agent effectively reduces systemic protein levels and mitigates amyloid fibril deposition in affected tissues [138,139].

Inotersen is a “chimeric MOE (2′-O-methoxyethyl)/DNA gapmer,” representing a sophisticated molecular design engineered to silence specific genes by targeting and degrading their mRNA, thereby inhibiting the synthesis of pathogenic proteins [140]. The molecule is a hybrid structure featuring a central DNA segment flanked by modified RNA sequences. This design enhances stability and target affinity, enabling durable protein knockdown via weekly dosing [141]. The DNA gap domain binds to target mRNA, forming a complex cleaved by RNase H to achieve gene silencing. Flanking “wings” enhance molecular stability and binding affinity, enabling sustained knockdown of both mutant and wild-type protein expression [142]. Moreover, they preclude intracellular nuclease degradation, thereby extending the therapeutic half-life and enabling once-weekly administration regimens [141].

The pivotal NEURO-TTR trial was a phase 3, randomized, double-blind study of 172 adults with ATTRv-PN, in which inotersen produced significant reductions in serum TTR (~70–80%) and meaningful improvements versus placebo in the modified mNIS+7 and the Norfolk QOL-DN scores at 66 weeks, with benefits maintained in the open-label extension [143].

Inotersen-induced TTR reduction improves neurological outcomes and quality of life; however, it requires strict monitoring for thrombocytopenia and glomerulonephritis [144]. Routine hematologic and renal monitoring is essential to manage immune-mediated risks. The standard weekly 284 mg dose of inotersen should be reduced to 150 mg or suspended if thrombocytopenia occurs. No adjustments are needed for elderly patients or those with renal impairment. Monitoring platelet counts and vitamin A levels is required, as inotersen reduces serum vitamin A. Adhering to safety protocols is required for a favorable benefit-risk profile for PN patients [145]. The clinical use and commercial availability of inotersen have declined with the introduction of newer TTR-silencing therapies, and its current availability varies by jurisdiction.

### 9.2. Eplontersen

Eplontersen is an ASO conjugated to a GalNAc3 moiety, designed to target TTR mRNA within hepatocytes. By initiating RNase H-mediated degradation of TTR mRNA, the agent suppresses hepatic synthesis of both TTRwt and TTRv [146]. Eplontersen is indicated for the treatment of adults with ATTRv-PN, representing the first therapeutic in its class administered via monthly SC injection (45 mg) [146].

In the NEURO-TTRansform trial of 144 adults, eplontersen showed efficacy for ATTRv-PN. By week 65, it reduced serum TTR levels by 81.7%, significantly outperforming the historical placebo group [147]. Treatment halted neuropathic progression and improved quality of life. Secondary analyses showed benefits for autonomic dysfunction, which worsened without treatment. Furthermore, eplontersen demonstrated a superior benefit-risk profile and required less monitoring than inotersen [147]. Switching from inotersen to eplontersen further reduced TTR, restored platelets, and enhanced tolerability. In the NEURO-TTRansform CM subgroup, 65 weeks of eplontersen improved LVEF and stroke volume compared to placebo, while other echocardiographic measures remained stable [148].

The CARDIO-TTRansform Phase 3 study evaluated eplontersen in 1432 participants with ATTRwt-CM or ATTRv-CM receiving standard of care. Patients were randomized to receive 45 mg of eplontersen or a placebo every 4 weeks. The primary endpoint was a composite of cardiovascular mortality and recurrent clinical events through week 140. Results showed that adding eplontersen to standard of care did not significantly reduce the primary endpoint risk [149].

Eplontersen is generally well-tolerated. Common side effects include injection site reactions, joint pain, fatigue, headache, and elevated liver enzymes. Daily vitamin A supplementation is required. Serious adverse events are rare.

## 10. Gene Editing

**Figure 9:**
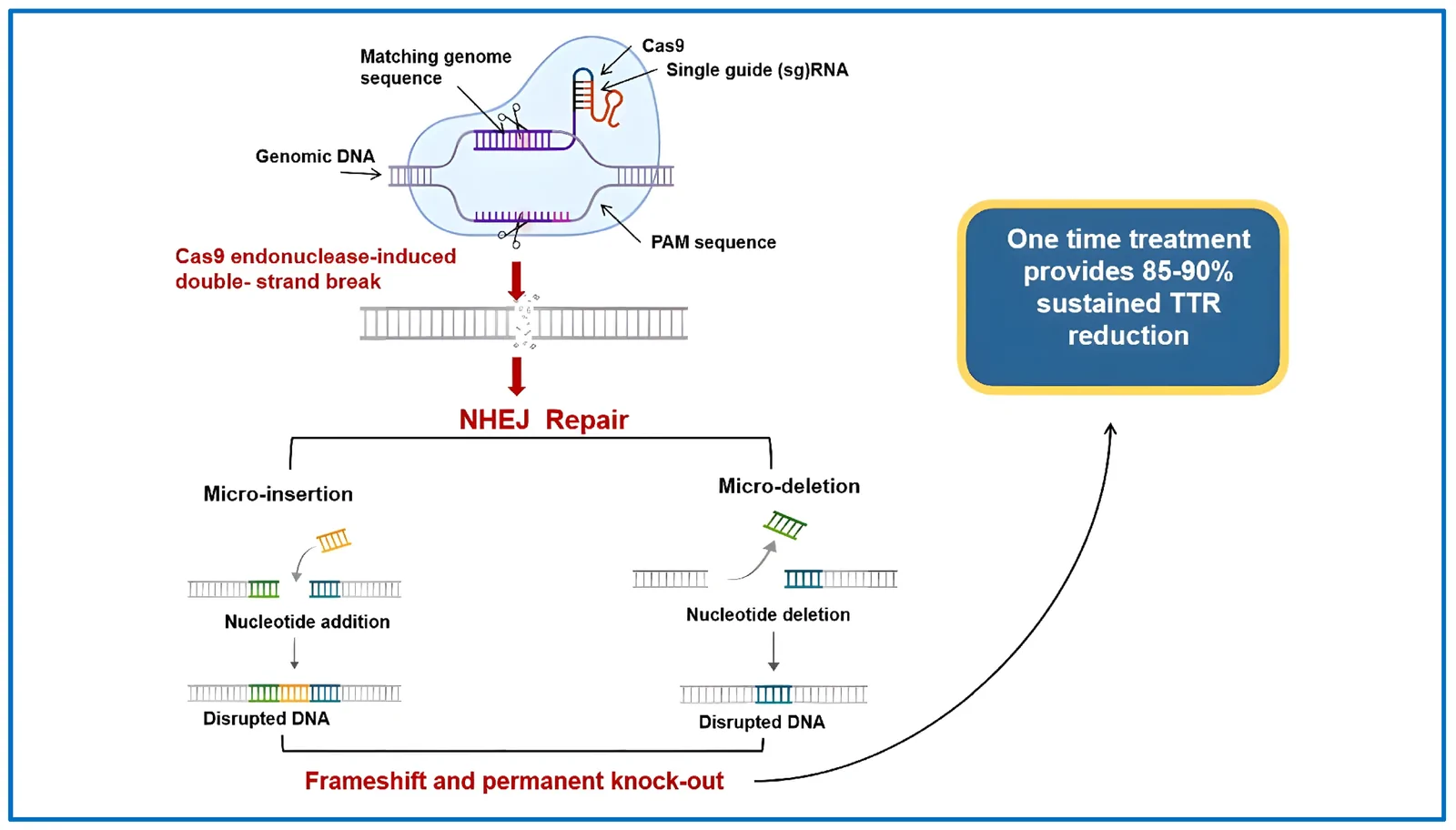
Schematic of a single-dose in vivo CRISPR–Cas9 strategy to reduce transthyretin (TTR) expression. Top: A Cas9-sgRNA complex targets the TTR locus to induce a double-strand break (DSB). Middle: non-homologous end joining (NHEJ) repairs the DSB, creating small insertions or deletions (indels). Bottom: these indels cause frameshifts or stop codons, resulting in permanent gene knockout. Right: intravenous administration leads to a durable ~85–90% reduction in circulating TTR. Cas9, CRISPR-associated protein 9; sgRNA, single guide RNA; PAM, protospacer adjacent motif; DSB, double-strand break; NHEJ, non-homologous end joining; TTR, transthyretin. Created in BioRender. Triposkiadis, F. (2026) https://BioRender.com/kuzq5ui.

Gene editing offers a durable therapeutic approach for ATTR by permanently inactivating the hepatic TTR gene. The CRISPR-Cas9 (Clustered Regularly Interspaced Short Palindromic Repeats/CRISPR-associated protein 9) system utilizes a programmable endonuclease and a single-guide RNA (sgRNA) to induce targeted DNA double-strand breaks (DSBs) (Figure 9) [150].

In the context of ATTR, the sgRNA is designed to target exon 2 of the TTR gene—a region conserved across both wild-type and variant alleles—thereby ensuring the comprehensive disruption of all transcripts [150]. Administered intravenously via LNPs, the Cas9 mRNA and sgRNA are delivered into hepatocytes [151]. Following translation, Cas9 complexes with sgRNA in the nucleus to edit the TTR locus. The resulting DSBs are repaired via the NHEJ pathway, introducing indels that cause frameshift mutations and permanent gene knockouts [152]. Given that NHEJ is the predominant repair mechanism in non-dividing hepatocytes, contemporary in vivo strategies prioritize gene disruption over precise sequence correction.

Nexiguran ziclumeran (nex-z; NTLA-2001), a CRISPR-Cas9 therapy, employs LNPs to deliver SpCas9 mRNA and an sgRNA targeting the TTR gene. In patients diagnosed with ATTRv-PN, a single IV infusion reduced serum TTR levels by more than 90% within 28 days [153]. In a trial of 36 patients with ATTR-CM, serum TTR concentrations fell by 89% at 28 days and remained 90% lower at 12 months, with 92% of participants showing improved or stable NYHA functional class [154]. Adverse events occurred in 34 patients: five had transient infusion-related reactions, and two showed treatment-related liver enzyme elevations. Serious adverse events, mostly related to underlying ATTR-CM, were reported in 14 patients.

On 27 October 2025, enrollment in the NTLA-2001 evaluating trials of MAGNITUDE (Phase 3 study for adults with ATTR-CM) and MAGNITUDE-2 (Phase 3 study for adults with ATTR-PN) was suspended following the identification of a patient exhibiting Grade 4 liver transaminase elevations accompanied by elevated bilirubin levels. Subsequently, on 29 October 2025, the FDA issued a verbal notification placing both trials on a formal clinical hold. In early 2026, the FDA lifted the hold following a consensus on the implementation of enhanced liver-safety monitoring protocols and revised exclusion criteria.

Several next-generation approaches aim to broaden the therapeutic window beyond DSB-inducing SpCas9. Beyond the structural genomic variations caused by DSBs at the target locus, a primary safety concern of conventional SpCas9 is off-target cleavage. Standard gRNAs can tolerate mismatches, causing Cas9 to cleave unintended genomic sites [155]. These off-target breaks can lead to oncogenic mutations if repaired incorrectly. For younger patients on long-term therapy, rare editing errors in hepatocytes pose cumulative genotoxic risks. To mitigate these dangers, high-fidelity Cas9 variants and next-generation technologies have been developed to avoid double-strand breaks and improve genomic safety [156]. These include:

### 10.1. Base Editing

**Figure 10:**
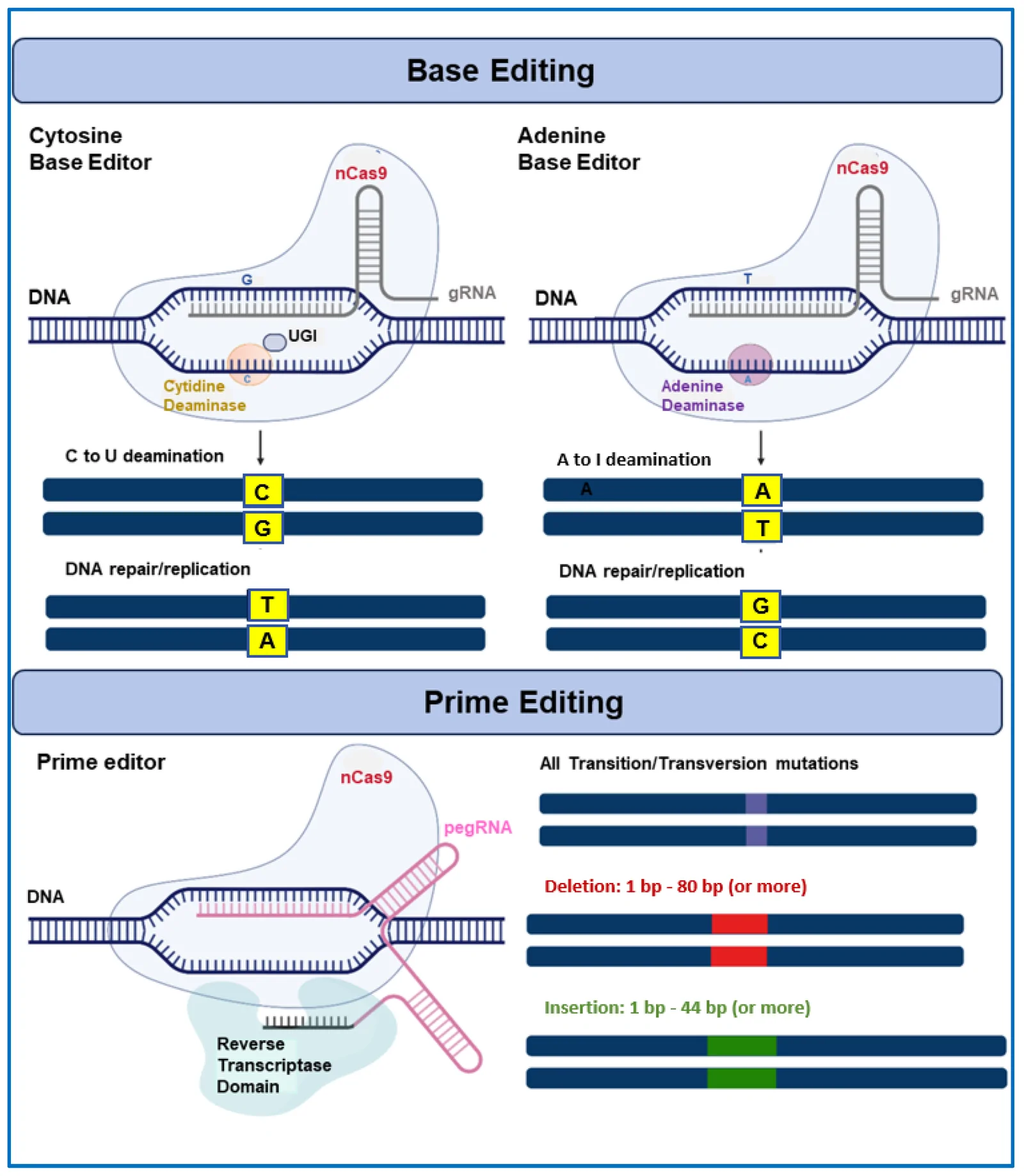
Mechanisms and capabilities of base editing and prime editing. Top Panel: Base Editing. Schematic illustration of transition mutation induction in the absence of double-strand breaks (DSBs). Cytosine Base Editor (CBE): a nickase Cas9 (nCas9) conjugated with a cytidine deaminase and a Uracil Glycosylase Inhibitor (UGI) uses a gRNA for locus-specific targeting. The deaminase facilitates the conversion of cytosine (C) to uracil (U) within the R-loop, while the UGI suppresses enzymatic repair. Subsequent DNA replication converts uracil into thymine (T), resulting in a C·G to T·A transition. Adenine Base Editor (ABE): An nCas9 fused to an adenine deaminase mediates the conversion of adenine (A) to inosine (I). Because DNA polymerases recognize inosine as guanine (G), this mechanism effects a targeted A·T to G·C transition. Bottom Panel: Prime Editing (PE). Schematic representation of the search-and-replace genome editing framework. Prime Editor Complex: An nCas9 fused to a reverse transcriptase (RT) is guided by a prime editing guide RNA (pegRNA). The pegRNA comprises a spacer sequence, a Primer Binding Site (PBS), and a Reverse Transcription Template (RTT) harboring the specified edit. Mechanism: The nCas9 induces a single-strand nick, enabling the liberated DNA strand to hybridize with the PBS. The RT subsequently synthesizes nascent DNA based on the RTT, thereby incorporating the desired modification. Endogenous cellular repair systems ultimately integrate the edit into both DNA strands. Created in BioRender. Triposkiadis, F. (2026) https://BioRender.com/icrllr9.

Cytosine base editors (CBEs) and adenine base editors (ABEs) consist of a Cas9 nickase fused to a deaminase that chemically converts C·G to T·A or A·T to G·C without introducing a DSB (Figure 10) [157,158]. This mechanism avoids the DNA repair cascades that drive DSB-associated genotoxicity and can be used to install nonsense codons or disrupt splice sites, splice acceptors, and 5′ splice donors within the TTR locus.

### 10.2. Prime Editing (PE)

It uses a Cas9(H840A) nickase fused to an engineered reverse transcriptase and a prime editing guide RNA (pegRNA) that simultaneously specifies the target site and encodes the intended edit [159] (Figure 10). The 3′ nicked DNA strand is reverse-transcribed using the pegRNA template, enabling precise insertions, deletions, and all base substitutions without a DSB. Prime editing systems like PE3, PE7, and engineered pegRNAs have further enhanced efficiency in primary cells [160,161]. TTR is a tractable prime-editing target because single amino-acid substitutions (e.g., the amyloidogenic V30M, T60A, V122I variants) can be corrected, although delivery to the liver in vivo remains the main translational hurdle [159,162].

### 10.3. Cas13 Nucleases

They are RNA-guided RNases that bind and cleave single-stranded RNA in a sequence-specific manner, with no requirement for, or modification of, the genome [163]. Hepatocyte TTR mRNA depletion via Cas13 enables a potentially reversible knockdown upon effector clearance. This offers a safety advantage over permanent DNA editing and avoids bystander damage from double-strand breaks. However, therapeutic suppression is transient, requiring periodic re-administration similar to siRNA-based drugs like patisiran and vutrisiran [126,164].

### 10.4. Epigenetic Editing

Catalytically dead Cas9 (dCas9) fused to a transcriptional repressor (e.g., KRAB) can silence TTR expression by depositing repressive chromatin marks at the promoter [165]. The unaltered genome allows gene expression to restore upon effector loss, providing a tunable and reversible suppression modality. This makes epigenetic editing a viable alternative when permanent gene knockout is unsafe, though the durability of repression and in vivo immunogenicity of dCas9-KRAB effectors require further study [166].

## 11. ATTR Treatment—Future Perspectives

The ATTR therapeutic landscape is shifting toward multi-mechanistic, precision interventions. Four emerging pillars—next-generation core therapies, fibril depleters, in vivo gene editing, and novel strategic paradigms—are redefining the management of ATTR-CM and ATTR-PN (Graphical Abstract). Together, these approaches move the field beyond chronic monotherapy toward deeper disease modification: robust biomarker suppression, histological regression, and, ultimately, durable genetic correction.

### 11.1. Next-Generation Core Therapies

Current management focuses on TTR stabilizers and RNA silencers. Acoramidis achieves near-complete TTR stabilization and showed clinical benefit in the Phase 3 ATTRibute-CM program, with follow-up supporting its durability and safety. Randomized head-to-head outcome superiority versus tafamidis is not established [167]. Vutrisiran reduced the risk of the primary composite of death or recurrent cardiovascular events in the HELIOS-B outcomes program and is an effective, quarterly-dosed RNA-silencing option for ATTR-CM [168]. Combination therapy is best framed by the step each drug class does not cover. Stabilizers preserve circulating tetramer and RBP transport but do not lower TTR production or remove deposited fibrils. Silencers and hepatic gene editing lower circulating TTR (and therefore vitamin A–RBP) but leave extrahepatic synthesis and existing tissue amyloid intact. Anti-fibril antibodies are designed to opsonize deposits and recruit macrophage clearance; they do not stabilize tetramers or silence the gene. The testable hypotheses are therefore: (i) silencer or editor plus stabilizer, to limit residual monomer from incompletely suppressed hepatic output; (ii) production blockade plus a depleter, to stop new deposition while clearing established Type A/B fibrils; and (iii) phenotype-specific sequencing (neuropathy-dominant versus cardiomyopathy-dominant versus leptomeningeal). Until factorial or add-on trials report hard endpoints—mortality, cardiovascular hospitalization, NIS-LL or 6-min walk, and imaging/biomarker regression—combination use should be described as investigational, not as an emerging standard.

### 11.2. Active Fibril Removal—The “Depleters”

Because stabilizers and silencers do not directly remove established tissue amyloid, amyloid-depleting monoclonal antibodies are being developed to clear fibrils and stimulate macrophage-mediated phagocytosis [169]. NI006 (ALXN2220) advanced into Phase 3 after Phase 1/1B and extension data showed dose-dependent reductions in tracer uptake and extracellular volume [170]. Coramitug (PRX004) reported Phase 2 biomarker and imaging improvements (notably at 60 mg/kg), received FDA Fast Track designation, and is being tested in the Phase 3 CLEOPATTRA study; definitive evidence of reduced mortality and hospitalization awaits ongoing Phase 3 results [171].

### 11.3. In Vivo Gene Editing

Single-dose, durable reduction in hepatic TTR is an aspirational curative strategy. NTLA-2001 (nex-z) SpCas9 LNP programs showed profound TTR knockdown in early work. Nevertheless, these clinical programs were briefly suspended in late 2025 due to safety concerns but resumed following FDA interactions, protocol amendments, and enhanced monitoring. Because DSB-inducing nucleases pose genotoxicity risks, alternative platforms such as base editing, prime editing, and CRISPRi are being investigated, each requiring trade-offs between precision, durability, and delivery.

### 11.4. Novel Strategic Paradigms

Three emerging trial paradigms are: (1) biomarker-guided enrollment with adaptive endpoints to accelerate readouts; (2) combination or sequential therapies targeting complementary biology, requiring careful safety and benefit evaluation; and (3) pre-symptomatic intervention in variant carriers to prevent phenotype emergence, which presents unique ethical and monitoring challenges. Long-term registries will be essential for all strategies.

## 12. Critical Unresolved Questions

Despite significant advancements, the clinical management of ATTR amyloidosis continues to encounter substantial challenges, with several critical questions still unresolved. A primary concern is the persistent difficulty associated with early detection, which complicates timely intervention. Furthermore, the comparative analysis of therapeutic modalities requires further investigation. It is essential to evaluate the relative efficacy and safety profiles of emerging therapies, such as gene-silencing agents and kinetic stabilizers, to identify optimal treatment strategies tailored to diverse patient phenotypes.

Long-term clinical outcomes also require rigorous examination. The enduring effects of current pharmacological interventions on quality of life and survival rates require elucidation through longitudinal studies to assess the durability of therapeutic benefits. Additionally, the potential efficacy of combination therapies warrants exploration; specifically, whether the integration of distinct therapeutic approaches can synergistically enhance patient outcomes remains an open question, the resolution of which could lead to more effective treatment paradigms.

Economic sustainability remains unresolved and should be stated with the evidence that actually exists. In a U.S. cost-effectiveness analysis of tafamidis for ATTR-CM was estimated at a list price that placed it among the most expensive cardiovascular drugs then marketed, with incremental cost-effectiveness ratios far above conventional willingness-to-pay thresholds unless substantial discounts were assumed [172]. A Spanish ATTR-PN costing study found that annual drug acquisition dominated total care costs: €291,076 for inotersen, €427,250 for patisiran, and €129,737 for tafamidis per patient-year [173]. Generic or repurposed kinetic stabilizers (diflunisal, tolcapone) are inexpensive by comparison but lack regulatory approval for ATTR and have narrower or less contemporary outcome data. These figures are list or study-specific acquisition costs, not net prices after rebate, outcomes-based contracts, or national negotiation, and they predate or only partly capture acoramidis, vutrisiran for cardiomyopathy, eplontersen, and one-time gene editing. Because approved disease-modifying agents are intended for long-term—often lifelong—use and are most effective when started early, list price, duration, monitoring burden, and vitamin A supplementation all affect real-world access. Comparative cost-effectiveness across stabilizers, silencers, editors, and future depleters, together with equity of reimbursement, therefore deserves dedicated analysis rather than a single rank-ordering sentence.

## 13. Conclusions

Therapeutic advancements have transitioned ATTR from a relentless, progressive disease with limited therapeutic options to an increasingly treatable disorder with multiple disease-modifying therapies. Kinetic stabilizers, RNA silencers, amyloid-depleting antibodies, and emerging gene-editing platforms each present distinct clinical advantages and limitations. Current academic and clinical priorities include early diagnosis, phenotype- and genotype-guided individualized therapy, rigorous long-term safety surveillance—particularly regarding gene-editing modalities—and the implementation of robustly designed combination and preventive clinical trials. The integration of structural biology with precision delivery systems is anticipated to facilitate the development of increasingly durable and, ultimately, curative therapeutic strategies.

## Data Availability

No new data were created or analyzed in this study. Data sharing is not applicable to this article.
